# Identification and characterization of proteins of unknown function (PUFs) in *Clostridium thermocellum* DSM 1313 strains as potential genetic engineering targets

**DOI:** 10.1186/s13068-021-01964-4

**Published:** 2021-05-10

**Authors:** Suresh Poudel, Alexander L. Cope, Kaela B. O’Dell, Adam M. Guss, Hyeongmin Seo, Cong T. Trinh, Robert L. Hettich

**Affiliations:** 1 Biosciences Division, Oak Ridge National Laboratory, Oak Ridge, TN 37831 USA; 2 The Center for Bioenergy Innovation at Oak Ridge National Laboratory, Oak Ridge, TN USA; 3 The Graduate School of Genome Science and Technology, University of Tennessee, Knoxville, TN USA; 4 The Bredesen Center, University of Tennessee, Knoxville, TN USA; 5 Department of Chemical and Biomolecular Engineering, University of Tennessee, Knoxville, TN USA

## Abstract

**Background:**

Mass spectrometry-based proteomics can identify and quantify thousands of proteins from individual microbial species, but a significant percentage of these proteins are unannotated and hence classified as proteins of unknown function (PUFs). Due to the difficulty in extracting meaningful metabolic information, PUFs are often overlooked or discarded during data analysis, even though they might be critically important in functional activities, in particular for metabolic engineering research.

**Results:**

We optimized and employed a pipeline integrating various “guilt-by-association” (GBA) metrics, including differential expression and co-expression analyses of high-throughput mass spectrometry proteome data and phylogenetic coevolution analysis, and sequence homology-based approaches to determine putative functions for PUFs in *Clostridium thermocellum*. Our various analyses provided putative functional information for over 95% of the PUFs detected by mass spectrometry in a wild-type and/or an engineered strain of *C. thermocellum*. In particular, we validated a predicted acyltransferase PUF (WP_003519433.1) with functional activity towards 2-phenylethyl alcohol, consistent with our GBA and sequence homology-based predictions.

**Conclusions:**

This work demonstrates the value of leveraging sequence homology-based annotations with empirical evidence based on the concept of GBA to broadly predict putative functions for PUFs, opening avenues to further interrogation via targeted experiments.

**Supplementary Information:**

The online version contains supplementary material available at 10.1186/s13068-021-01964-4.

## Background

Lignocellulose solubilization and fermentation have been major challenges in the quest to produce cost-effective cellulosic biofuels. *Clostridium thermocellum* (which has also been renamed as *Ruminiclostridium thermocellum* [1]*, Hungateiclostridium thermocellum* [2]*, Acetivibrio thermocellus* [3]) is a fermentative anaerobic thermophile that has been studied extensively as a possible chassis organism for this goal. Several attempts have been made to engineer *C. thermocellum* strains to produce bioethanol as the major cellulose degradation product at high yield [4–8], but none of these attempts have matched conventional bioethanol producers, such as *Saccharomyces cerevisia*e and *Zymomonas mobilis* [9, 10].

Although *C. thermocellum* produces various short-chain alcohols (e.g., ethanol, isobutanol, etc.), several other end products are also generated (e.g., formic acid, acetic acid, lactic acid, hydrogen, amino acids, etc.). In particular, the organic acids decrease pH of the culture media and reduce yields of alcohols as biofuels. To improve ethanol production, a modified version of *C. thermocellum* DSM1313 was generated, called strain LL1210, in which the specific genes involved in the production of acetate, lactate, formate, and most hydrogen (*Δhpt ΔhydG Δldh Δpfl Δpta-ack*) have been deleted, followed by adaptive laboratory evolution strategy [11]. While LL1210 is among the highest producers of ethanol titer and yield from lignocellulosic biomass, further advances in this strain are required to examine organism robustness and scalability for industrial applications [10].

Interestingly, many of the proteins determined to be differentially expressed or highly expressed based on specific substrate in *C. thermocellum* and other cellulolytic bacteria are annotated as hypothetical proteins, uncharacterized proteins, domains of unknown function (DUFs), or a similar term indicating no known function. We broadly refer to this class of proteins as “proteins of unknown function” (PUFs). High abundances and/or differential expression of PUFs that are sensitive to environmental conditions (specifically, cellulosic substrate type) suggests a possible role in the metabolism of cellulose or other key cellular processes. For example, previous work in the cellulolytic *Caldicellulosiruptor bescii* indicated differential abundance of 37 PUFs driven by the nature of the cellulosic substrates used in the growth media [12]. Similarly, many PUFs were found to be highly and/or differentially abundant across four strains of *C. thermocellum* (one wild-type parent strain plus three mutant strains) [11]. For example, WP_003519067.1 (Clo1313_1790), which was a PUF at the time of this study, was highly abundant across the 4 strains [11], suggesting an important functional role even in mutants that had undergone adaptive laboratory evolution. WP_003519067.1 is now annotated in NCBI RefSeq as a 2Fe-2S ferredoxin based on a conserved domain identified by NCBI SPARCLE [13]. Some PUFs were highly abundant in mutants, but not in the wild-type strain, while other PUFs showed differential abundance across mutant strains. Such measurements suggest a functional role, but a key challenge for researchers is to identify the specific function of a PUF.

As evident from the critical assessment of protein function annotation (CAFA), functional predictions based on sequence homology have dramatically improved over the past 2 decades [14–16]. Despite this progress, a large number of proteins remain annotated as PUFs [17]. As of March 2020, a total of 17,929 domains were deposited in the Pfam database, with 5792 domains (32% of the total) containing the keyword “unknown function” [18]. Reports indicate that a large fraction of Protein Data Bank (PDB) entries are categorized under “unknown functions” [19, 20]. PUFs are common even in well-studied species. For example, only 40% of predicted genes in the model plant *Arabidopsis thaliana* have reliable annotations [21]. Previous efforts have been made to predict the biochemical functions for protein structures of unknown function [22] and to characterize essential domains of unknown function (DUFs) [23]. Even after a recent attempt to better annotate  PUFs in the *S. cerevisiae* and human genomes via sequence homology, greater than 30% of their unknown proteins (600 and 2000 proteins, respectively) remain uncharacterized [24]. In *E. coli*, 80% of predicted proteins have some functional annotation, but only 54% have some level of empirical characterization [25, 26].

Protein characterization via empirical methods is challenging due to a large amount of sequencing data currently available combined with the low-throughput nature of characterization experiments. An alternative approach is to use interaction or co-expression data produced via high-throughput omics-scale measurements to identify proteins of known functions with which a PUF is associated, a concept referred to as “guilt-by-association” (GBA) [24, 27–31]. GBA operates under the reasonable assumption if two proteins physically interact or are co-expressed with one another, they are more likely to be connected in function [29]. Previous work has found significant overlap between co-expression and protein–protein interaction networks, suggesting that functionally related proteins are co-expressed [32]. Using the concept of GBA, PUFs which interact or co-express with proteins of known function may serve similar functional roles as the former, which can be confirmed via targeted characterization experiments.

Given that approximately 20% of the *C. thermocellum* genome consists of PUFs, the goal of this work is to identify putative functional roles for PUFs in *C. thermocellum*, with a focus on PUFs which may play a role in cellulose degradation and ethanol production. To explain the potential roles of these PUFs, a time-course MS-based proteomics study was performed with *C. thermocellum* DSM1313 wild-type (∆hpt) and the evolved LL1210 strain to assess differential and co-expressed PUFs. ∆hpt is the parent strain for essentially every mutant ever made in *C. thermocellum*. It has a deletion in the hypoxanthine phosphoribosyl transferase (hpt), which allows use of ∆hpt as a counter-selectable marker for making gene deletions. 

The LL1210 strain was chosen to compare with the ∆*hpt* wild-type strain not only because they are genetically and phenotypically distinct but also because LL1210 is the highest ethanol producing strain of *C. thermocellum* to date. Therefore, discovery of PUFs within the strain could lead to advances in improving its metabolism toward ethanol production as well as overall growth .

The major aim of this experimental design was to explore the temporal response of PUFs that are specific to a particular strain and more importantly increase or accumulate along with the soubilization of the substrate. GBA evidence was leveraged with various functional prediction tools, structural modeling, phylogenetic analysis, and gene regulatory information to propose putative functional roles for many PUFs in *C. thermocellum*. In an attempt to validate functional predictions derived here, PUF candidates which could be tested and verified by a measurable phenotype effect, either *in vitro* or *in vivo*, were identified. This is a very difficult and unpredictable process with the risk of no positive return. A range of PUFs were considered and the best validation candidate selected. From this, PUF WP_003519433.1 was empirically validated, showing clear evidence to support the alcohol acetyltransferase activity prediction.

## Results

**Fig. 1:**
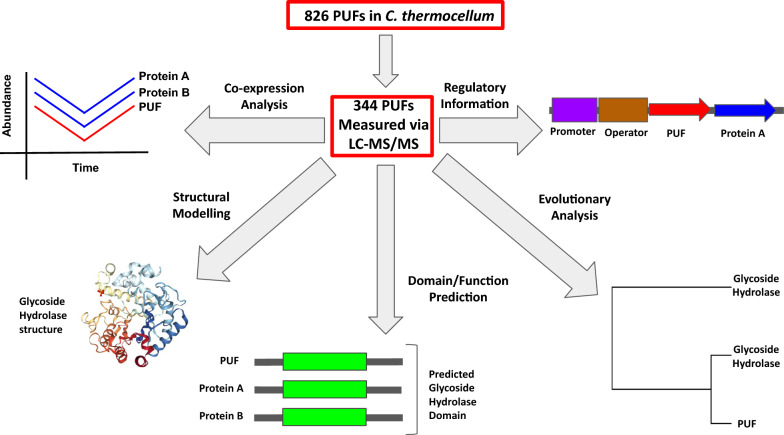
A pipeline summarizing the guilt-by-association and functional annotation approaches used in this study. The 344 PUFs measured via LC–MS/MS were subjected to co-expression and differential expression analyses. Structural modeling with SwissModel was used to determine structural templates which best fit a PUFs protein sequence. Domain and function prediction were performed using InterProScan, eggNOG-mapper, and PANNZER2. Phylogenetic coevolution analysis was used to test for coevolution. Gene trees were generated from a homology search in BLAST, followed by alignment of the top 200 hits and tree construction using FastTree. Regulatory information based on shared operons was extracted from the DOOR database

A visual outline of the GBA approach described in this manuscript is presented in Fig. 1, which illustrates how the MS-based proteome information is first connected with expression networks and then interrogated with a variety of informatics and structural prediction tools. PUFs with consistent lines of evidence across multiple GBA approaches were deemed strong candidates for putative functional classification.

**Fig. 2:**
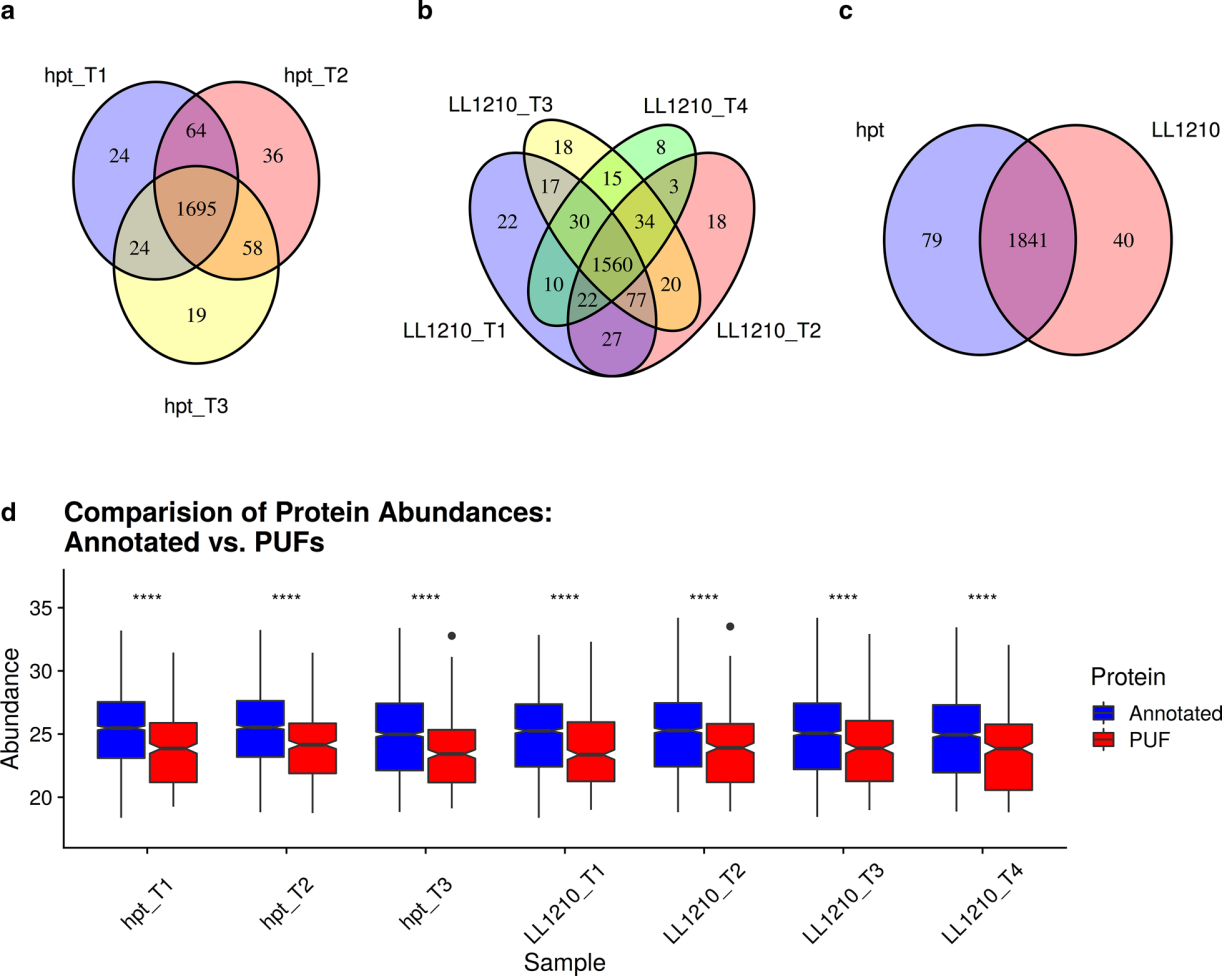
Summary of protein identifications and abundances. Overlap of protein identifications across time points in **a** *Δhpt* and **b** LL1210, and **c** overlap between strains. **d** Distribution of protein abundances for annotated proteins and PUFs across all strains and time points. ****p <  = 0.0001.31

A total of 1960 proteins out of 3033 possible proteins (65%) were quantified across all time points (as defined in "Methods" section) in both *C. thermocellum* strains (*∆hpt* and LL1210).  Figure 2 demonstrates the global proteome overlap (Venn-diagram) across both strains (a–c) and distribution of protein abundances (annotated versus PUFs), as shown by boxplots (d). In both strains, each time point had several unique proteins (Fig. 2a and b); however, a majority of proteins were observed in both experimental strains (Fig. 2c). This reveals that while much of the overall protein machinery is constant, some of the identified proteins are specific for one strain under the provided growth condition, which could help to characterize and understand the overall functionality of that strain. The unique proteins in the *∆hpt* strain were enriched in function related to sulfur compound metabolic process (GO:0044272), drug metabolic process (GO:0017144), oxidation–reduction process (GO:0055114), aromatic compound biosynthetic process (GO:0019438), and water-soluble vitamin biosynthetic process (GO:0042364). Notably, many proteins involved in these processes are perturbed in the LL1210 strain [11]. In contrast, the unique proteins in the LL1210 strain were related to polysaccharide catabolic process (GO:0000272). Global abundance distribution between all annotated identified proteins versus PUFs revealed that PUFs are, on average, lower in abundance across all conditions (Fig. 2d); however, since they are identified, they likely play key roles in the solubilization of the substrates.

In total, 344 PUFs were identified via LC–MS/MS and were interrogated with GBA and sequence homology-based analyses. Across all time points, proteins with functional annotations were on average of higher abundance, as expected, although some PUFs are clearly highly abundant (Fig. 2d). At the time of our experiment, PUFs WP_003518117.1 and WP_003519055.1 were the only PUFs found in the top 10% most abundant protein across all samples. However, these PUFs were recently annotated as Ig-like domain containing protein and (2Fe-2S) ferredoxin domain-containing protein, respectively. The results section will focus on some broad trends observed related to PUFs (e.g., differential expression patterns, coevolution patterns, etc.) followed by the description of specific PUFs of interest.

### Phylogenetic analysis reveals PUFs coevolving with cellulose solubilization and cellulosome structural proteins

To reduce ambiguity in unique protein assignments, orthogroups, or sets of orthologous and paralogous proteins across species, were determined using the software OrthoFinder [33] with 27 species that included both cellulolytic and non-cellulolytic Firmicutes. Of the 344 PUFs detected via LC–MS/MS, 68 were assigned to 37 unique orthogroups. To examine coevolution of PUFs with other proteins, we employed a phylogenetic method that tests for correlated presence or absence of traits across species [34]. The traits in this case were the orthogroups, specifically the 37 containing a PUF measured via LC–MS/MS. Using the species tree estimated by OrthoFinder (Additional file 1: Figure S2), our analysis detected 115 PUFs that exhibited significant signals of coevolution with another protein orthogroup. Interestingly, 76 of the 115 significant results indicated PUFs that coevolved with WP_003516608.1 (YcxB family protein), WP_003516626.1 (zinc-finger transcription factor II domain-containing protein), or ADU74616.1 (PUF, not detected by LC–MS/MS). Although the function has not been characterized, YcxB family proteins are predicted to be transmembrane proteins. The set of proteins shown to be coevolving with PUFs were enriched in GO terms related to polysaccharide catabolic process (GO:0000272), chemotaxis (GO:0006935), cell wall macromolecule catabolic process (GO:0016998), xylan metabolic process (GO:0045491), transmembrane signaling receptor activity (GO:0004888), cellulose binding (GO:0030248), cellulose 1,4-beta-cellobiosidase activity (GO:0016162), calcium ion binding (GO:0005509), and O-glucosyl hydrolase activity (GO:0004553), among others, as shown in Additional file 2. Clearly, many of these processes are related to the solubilization of cellulose.

### Comparison of strains *Δhpt* and LL1210 reveals differential protein expression of both known and unknown (PUF) proteins.

Differential expression analysis of protein abundances was performed using limma [35] between the two strains at early-log phase, mid-log phase, and late-log phase. Results of various functional enrichments can be found in Additional files 3 and 4. In total, we found 707 unique proteins that were differentially expressed in at least one time point, 100 of which were PUFs. For each time point, there were 393, 414, and 444 differentially expressed proteins between the *∆hpt* and LL1210 strains, respectively. Of these, 57, 53, and 59 were PUFs, with 38, 27, and 37 of these having an absolute log2 fold change of at least 1.5 (see volcano plot, Additional file 5: Figure S3). Sets of differentially expressed proteins were enriched in various GO terms and KEGG terms at each time point (Additional files 3 and 4). Under the assumption of GBA, differentially expressed PUFs are likely to have similar functional roles.

During early-log and late-log phases, differentially expressed proteins were enriched in GO and KEGG terms related to flagellum-dependent cell movement (GO:0071973) and chemotaxis (GO:0006935). These biological processes appear to be overall up-regulated in LL1210 during early-log phase, with mean log2 fold changes of 0.24 and 1.03, respectively. However, these processes appear to be down-regulated in late-log growth (mean log2 fold change -2.16 and -1.75, respectively). In addition, gene set enrichment analysis of differentially expressed proteins revealed that proteins with KEGG terms related to flagellar assembly (KEGG ID ctx02040) were less abundant across all 3 time points in LL1210 relative to *∆hpt*. Previous work found that proteins related to cell motility were down-regulated in the LL1210 strain [10]. Cellular motility can be an energetically costly process, so the already slow-growing strain with a heavily perturbed proteome could down-regulate cellular motility processes to channel ATP to other key cellular processes, consistent with many of our observations. Note that sporulation genes, specifically the master regulator SpoA, was mutated in LL1210. In other clostridia species, mutations in *spoA* have affected biofilm and flagellum expression.

At all three time points, proteins involved in the acetyl-CoA biosynthesis (GO:0006085) also appear to be differentially expressed (i.e., 1.08, −2.18, −2.80 mean log2 fold change in early, mid-, and late-log phases, respectively). This result is consistent with the genetic modification of the LL1210 strain, which started as a strain with the pyruvate-formate lyase-dependent pathway converting pyruvate to acetyl-CoA disrupted. In addition, there is an overall increased abundance in LL1210 of proteins involved in the pantothenate metabolic processes (GO:0015939) at the mid-log phase (mean log2 fold change 1.97). This finding is particularly interesting as pantothenate is the precursor for CoA biosynthesis which has a range of functions in bacteria [36]. As the acetyl–CoA pathway has been altered in the LL1210 strain to drive the pyruvate metabolism towards ethanol production, this increased abundance of pantothenate metabolism could indicate changes to fatty acid metabolism. A previous study found that *C. thermocellum* adapts to the increase of ethanol by remodulation of the cell membrane [37]. Consistent with this conclusion, we found that proteins with GO:0006633 (fatty acid biosynthetic processes) and GO:0004312 (fatty acid synthase activity) were both more abundant in LL1210.

At all three time points, GO term GO:0016730 (oxidoreductase activity, acting on iron–sulfur proteins as donors) was more abundant in the LL1210 strain relative to *Δhpt* (mean log fold change 4.14, 2.56, and 2.44 in early, mid-, and late-log phases, respectively). The reduction of oxidized ferredoxin (an iron–sulfur protein) is an important step in the conversion of pyruvate to acetyl-CoA. Notably, GO:0008901 (ferredoxin hydrogenase activity) was also more abundant (mean log2 fold change 1.30) in LL1210 at mid-log phase.

Aside from identifying differentially expressed proteins at each time point, we also sought to identify differentially co-expressed proteins between the *Δhpt* and LL1210 strains. We identified 359 differentially co-expressed proteins between the *Δhpt* and LL1210 strain. Of these 359 proteins, 50 were PUFs. These differentially co-expressed proteins were enriched in GO terms related to dephosphorylation (GO:0016311), positive regulation of gene expression (GO:0010628), cell adhesion (GO:0007155), metal ion transport (GO:0030001), phosphate-containing compound metabolic processes (GO:0006796), cellular response to oxygen-containing compound (GO:1,901,701), magnesium ion binding (GO:0000287), cyclic-di-GMP binding (GO:0035438), transferase activity, transferring phosphorous containing groups (GO:0016772), and isomerase activity (GO:0016853), among others. As noted above, differential expression related to iron-binding proteins could be significant due to their role in the conversion of pyruvate and ethanol. We note that two PUFs with GO terms related to iron-ion binding were found to be differentially co-expressed: WP_003512015.1, which is discussed further below, and WP_003515910.1.

### Co-expression analysis

Co-expression analysis was performed separately for the *Δhpt* and LL1210 strains to determine clusters of co-expressed genes. Using the Python tool clust [38] we identified 11 and 14 clusters of co-expressed proteins in the *Δhpt* and LL1210 strains, respectively. The cluster-specific protein abundance patterns can be seen for these strains in Additional file 6: Figure S4 Additional file 7: Figure S5, respectively. In total, these clusters represented co-expression patterns of 1226 and 786 proteins in the *Δhpt* and LL1210 strains, respectively. Functional enrichment was performed to assess potential functions of PUFs based on GBA. Out of the numerous PUFS that were identified in this study, we will highlight a few below that are of particular interest due to their potential role in cellulose solubilization, pyruvate metabolism, and/or ethanol production.

### PUF WP_003512015.1 (Clo1313_2169): Evidence for a rubredoxin protein

**Fig. 3:**
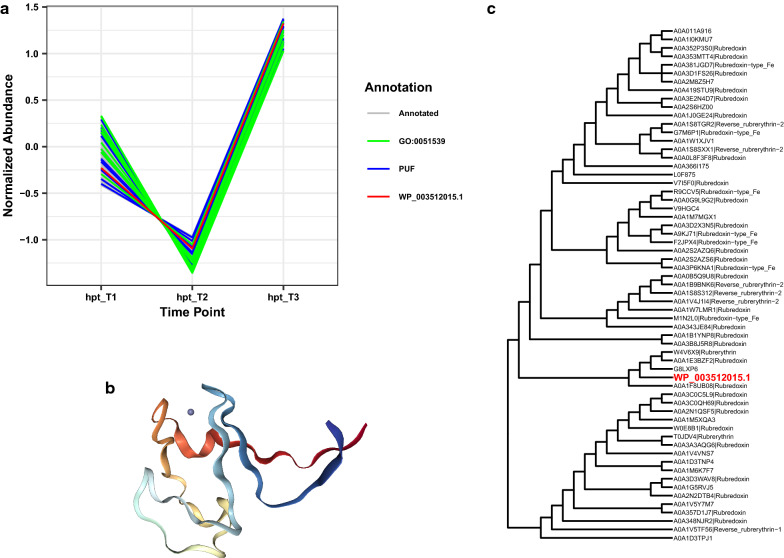
Results for possible rubredoxin, PUF WP_003512015.1. **a** Co-expression cluster of PUF WP_003512015.1, including other PUFs and proteins with GO:0051539 (4 iron, 4 sulfur cluster binding). **b** Best fitting structure of known function from PDB, 1H7V, which is a rubredoxin from *G. theta* (sequence similarity 0.42 and coverage 0.32). **c** Phylogenetic gene tree for WP_003512015.1 indicates that this protein is closely related to many rubredoxin proteins

GBA and sequence homology-based evidence suggest that WP_003512015.1 is a rubredoxin protein, a protein consisting of one iron atom that serves as an electron carrier. WP_003512015.1 was found in cluster hpt_C5 and LL1210_C7. Although LL1210_C7 contained no enriched GO or KEGG terms, hpt_C5 (Fig. 3a) was enriched in many functional terms, including 4 iron, 4 sulfur cluster binding (GO:0051539), metal ion binding (GO:0046872), and oxidoreductase activity, acting on the CH-OH group of donors and NAD or NADP as acceptor (GO:0016616). Interestingly, this PUF falls into clusters which qualitatively appear to demonstrate differential co-expression patterns between the *Δhpt* and LL1210 strain. In the *Δhpt* strain, WP_003512015.1 decreases from early-log to mid-log phase before a large jump in abundance in late-log phase. The opposite pattern is observed in LL1210, where there is a small increase in WP_003512015.1 from early-log to mid-log phase, followed by a sharp decrease into late-log phase. If this PUF is involved in the oxidation–reduction processes in the conversion of pyruvate to ethanol, then contrasting patterns between the *Δhpt* and LL1210 strain might be expected. WP_003512015.1 was differentially expressed between the two strains at late-log phase, with a log2 fold change of -1.87. If WP_003512015.1 has oxidoreductase activity, then this is consistent with its differential expression along with many other proteins with similar biological function. However, WP_003512015.1 was not significant based on our differential co-expression analysis (dCp = 0.3, q value = 0.065).

Sequence homology-based evidence strongly supports WP_003512015.1 as a rubredoxin. The best fitting structure from SwissModel is a rubredoxin protein found in *Guillardia theta* (PDB 1H7V, Fig. 3b), but many other structures were annotated as rubredoxins or rubredoxin-like proteins. Furthermore, examination of the phylogenetic gene tree reveals WP_003512015.1 is closely related to many rubredoxin proteins annotated in UniProt (Fig. 3c). Although the operon for WP_003512015.1 does not regulate expression for any other proteins, it is annotated as a rubredoxin-type protein in the DOOR database [39], consistent with the co-expression and homology-based analyses. This result also highlights the limitations of the RefSeq and GenBank repositories to reflect the most up-to-date functional annotations.

### PUF WP_003516357.1 (Clo1313_1439): Evidence for an ABC transporter

**Fig. 4:**
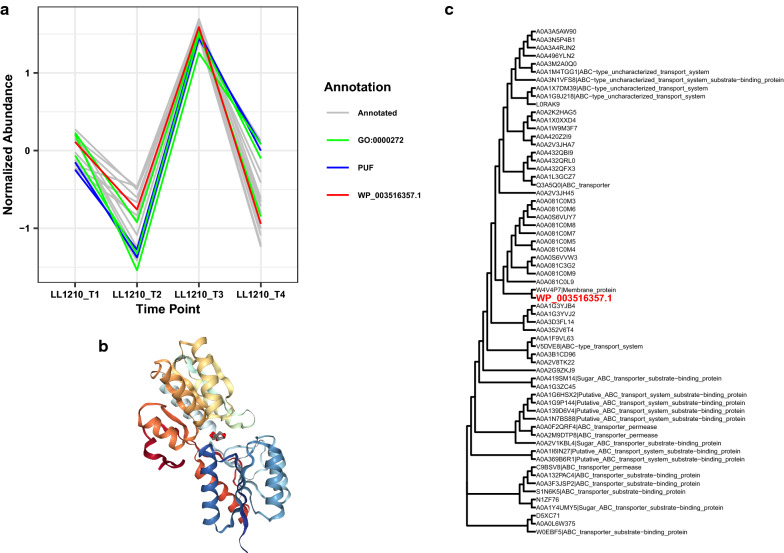
Results for possible ABC transporter, PUF WP_003516357.1. **a** Co-expression cluster of PUF WP_003516357.1, including other PUFs and proteins with GO:0000272 (polysaccharide catabolic process). **b** Best fitting structure of known function from PDB, 5IBQ, which is annotated as a probable ribose ABC transporter, substrate-binding protein (sequence similarity 0.27 and coverage 0.67). **c** Phylogenetic gene tree for WP_003516357.1, which is closely related to proteins related to ABC transport systems

Various lines of evidence suggest that PUF WP_003516357.1 is a component of a sugar ABC transporter. WP_003516357.1 is differentially expressed in at all 3 time points between the *Δhpt* and LL1210 strains, with log fold changes of -2.95, -3.31, and -4.20. This indicates relatively lower abundance of WP_003516357.1 in the LL1210 strain. WP_003516357.1 falls into LL1210_C13 (Fig. 4a), which consists of 17 proteins, 3 of which are PUFs. This network is enriched in GO terms chemotaxis (GO:0006935), polysaccharide catabolic process (GO:0000272), DNA-dependent DNA replication (GO:0,006,261), and carbohydrate binding (GO:0030246). Although none of the enriched GO terms directly relate to protein transport, sequence homology-based approached strongly suggests that this PUF is likely an ABC transporter. InterProScan [40] identifies WP_003516357.1 as an ABC transporter, substrate-binding protein. Furthermore, the vast majority of structural templates fitting to WP_003516357.1 come from sugar ABC transporters, consistent with the enrichment of polysaccharide catabolic process and carbohydrate-binding proteins in LL1210_C13. Consistent with this, this small cluster contains WP_003515342.1 (glycoside hydrolase), WP_003519375.1 (cell surface glycoprotein 2), and WP_014522595.l (cellulosome anchoring protein cohesin subunit). The best fitting structure of known function is annotated as a probable ribose ABC transporter, substrate-binding protein (PDB 5IBQ, Fig. 4b). Additionally, five of the matched structures were related to the transport of arabinose, a monosaccharide found in the hemicellulose of plant cell walls. We note that *C. thermocellum* is known to use ABC transporter systems for the uptake of oligosaccharides [37]. The gene tree supports this protein as a component of an ABC transporter. WP_003516357.1 is most closely related to a membrane protein, but ABC transporters and ABC-type uncharacterized transporters are also present in the gene tree (Fig. 4c). Taken all together, PUF WP_003516357.1 has strong evidence as a protein component of an ABC transporter possibly involved in the uptake of carbohydrates.

### PUF WP_003511984.1 (Clo1313_2180): Evidence for a glycoside hydrolase

During the process of our data analysis for this study, we focused attention on PUF WP_003511984.1, as we had strong GBA evidence that it was a glycoside hydrolase. Interestingly, in the most recent reannotation of the *C. thermocellum* genome, this protein is now labeled as a putative glycoside hydrolase. Since our examination of this protein was completed in the absence of that information, we hereby present below the evidence we had that converged on the same functional assignment as the reannotation, as a type of positive control for our PUF approach.

**Fig. 5:**
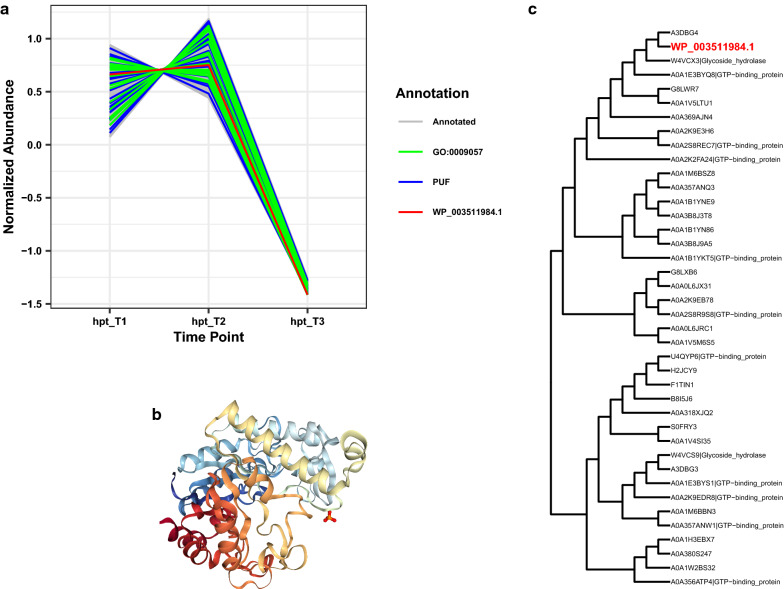
Results for possible glycoside hydrolase, PUF WP_003511984.1. **a** Co-expression cluster of PUF WP_003511984.1, including other PUFs and proteins with GO:0009057 (macromolecule catabolic process). **b** Best fitting structure of known function from PDB, 5OQ2, which is protein Cwp19 in *C. difficile* and contains a glycoside hydrolase domain (sequence similarity 0.28 and coverage 0.70). **c** Phylogenetic gene tree for WP_003511984.1 indicates this protein is closely related to a glycoside hydrolase, but many GTP-binding proteins are also present

WP_003511984.1 was not differentially expressed between the *Δhpt* and LL1210 strains at any of the time points; however, it was differentially co-expressed (dCp = 0.93, q value = 0.034). WP_003511984.1 was found in clusters hpt_C0 (Fig. 5a) and LL1210_C0, which are two large clusters with 205 and 167 genes, respectively. Both clusters had many enriched GO terms. The strongest evidence for WP_003511984.1 as a glycoside hydrolase was the enrichment of the GO term macromolecule catabolic process (GO:0009057). We note that this does not necessarily refer to polysaccharide catabolism. However, examination of the proteins with this GO term in the hpt_C0 cluster included proteins annotated as glycoside hydrolases (ADU75731.1, WP_003515281.1, WP_003517278.1), endoglucanase (WP_003512420.1, WP_003514472.1, WP_003517595.1), carbon storage regulators (WP_003513578.1), *N*-acetylmuramoyl-l-alanine amidase (a cell wall hydrolase, WP_003515629.1), carbohydrate-binding domain-containing protein (WP_003516871.1), glycosyl transferase (WP_003518177.1), and a copper–amine oxidase (WP_003518386.1), with GO terms related to polysaccharide catabolic process (GO:0000272), chitin binding (GO:0008061), and carbohydrate binding (GO:0030246).

Further examination of predicted protein structures also supports WP_003511984.1 as a glycoside hydrolase. Predicted structures include multiple beta-galactosidase structures, consistent with results of EGAD related to carbohydrate metabolism. The best matching structure of known function for WP_003511984.1 is annotated as Cwp19 (PDB 5OQ2). This protein is found in *Clostridium difficile*, and the structure represents the glycoside hydrolase domain of Cwp19 (Fig. 5b).

The phylogenetic gene tree also indicates WP_003511984.1 is similar in sequence to glycoside hydrolases (Fig. 5c). PANNZER2 [41] annotates this protein as a potential glycoside hydrolase. WP_003511984.1 was predicted to have a signal peptide and a transmembrane region. Taken together, current evidence strongly suggests that this protein is a glycoside hydrolase. As noted above, a recent reannotation in the RefSeq database established this as a putative glycoside hydrolase, consistent with the results presented here.

### PUF WP_003519433.1 (Clo1313_1074): Evidence and experimental validation as an alcohol acetyltransferase activity

**Fig. 6:**
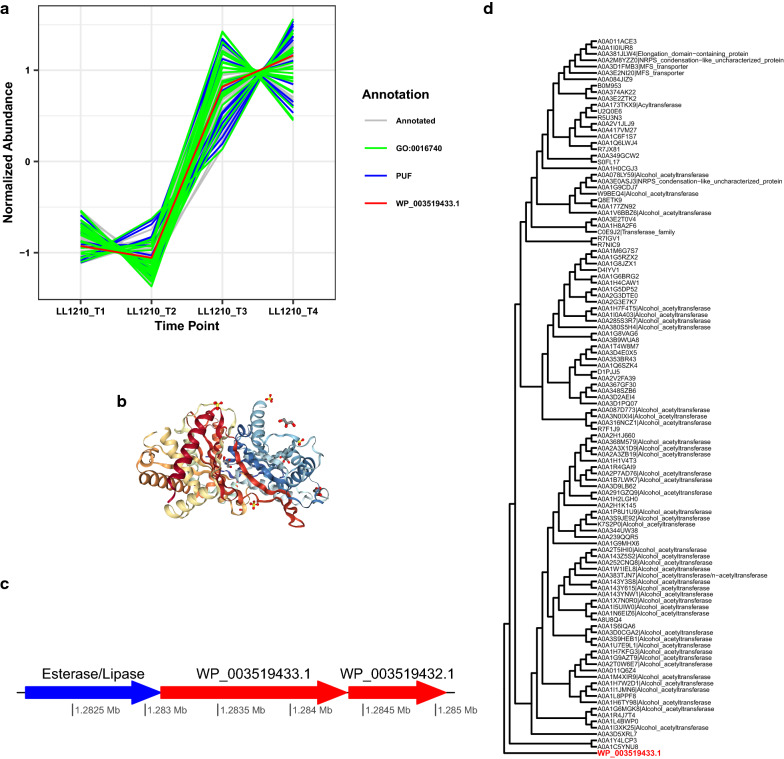
Results for possible alcohol acetyltransferase, PUF WP_003519433.1. **a** Co-expression cluster of PUF WP_003519433.1, including other PUFs and proteins with GO:0016740 (transferase activity). **b** Best fitting structure of known function from PDB, 3FOT, which is annotated as a 15-O-acetyltransferase (0.27 sequence similarity and 0.89 sequence coverage). **c** Cartoon representation of operon structure according to DOOR database. **d** Partial phylogenetic gene tree for WP_003519433.1, which is closely related to proteins related to alcohol acetyltransferase. The complete tree contains many proteins annotated as alcohol acetyltransferases aside from those seen in the partial tree

Exploring WP_003519433.1 at several levels such as annotation using PANNZER2, eggNOG-mapper [42], phylogenetic gene trees, and structural modeling all indicated that WP_003519433.1 is a probable alcohol acetyltransferase (Fig. 6). WP_003519433.1 was not found to be differentially expressed or differentially co-expressed between strains. WP_003519433.1 was found in hpt_C0 and LL1210_C1 clusters (Fig. 6a). The strongest co-expression evidence is the enrichment of GO term transferase activity (GO:0016740) in LL1210_C1. Although this is a broad GO term, we note that proteins falling into this cluster with this GO term could be an acetyltransferase as this cluster also includes an N-acetyltransferase (WP_003513195.1) and PUF WP_003513604.1 with GO term N-acetyltransferase activity. Cluster LL1210_C1 was also enriched for the KEGG module Shikimate pathway, which is responsible for the synthesis of folate and aromatic amino acids. Notably, PUF WP_003519433.1 has a GO term indicating that it is possibly a membrane protein and fits the structural template of a TRI3 Tricothecene 15-O-acetyltransferase from the fungus *Fusarium sporotrichioides* (PDB 3FOT). PUF WP_003519433.1 is also part of an operon, a key piece of GBA evidence, with a protein annotated in DOOR as an esterase/lipase (Fig. 6c). WP_003519433.1 was selected for further characterization. Interestingly, the phylogenetic gene tree appears to be split between two major groups: one in which many of the proteins are annotated as an alcohol acetyltransferase or similar function, and a group that is mostly PUFs (Fig. 6d).

**Fig. 7:**
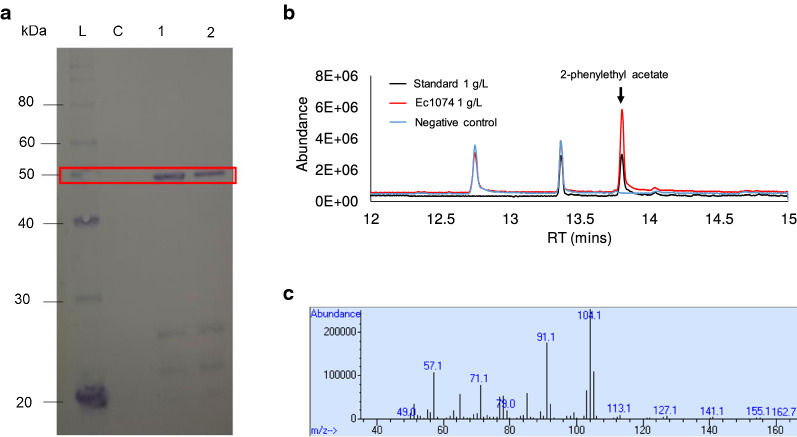
**a** Western blot of WP_003519433.1 expression in *E. coli*. L: protein ladder; C: protein purified from no IPTG-induced cells (negative control); Lane 1: protein purified from cells induced with 0.1 mM IPTG; and 2: protein purified from cells induced with 1 mM IPTG. The band signals observed in lanes 1 and 2 in the red box confirmed the identity of WP_003519433.1 with an expected protein size of 50.4 kDa. **b** Total ion chromatography of high cell density *E. coli* whole-cell conversion of 2-phenylethyl alcohol. *E. coli* harboring empty plasmid was used a negative control. **c** Mass-to-charge ratio of the selected 2-phenylethyl acetate peak. Here, the eluted peaks at the retention of 15.5 min in panel B confirmed that 2-phenylethyl acetate was produced by Ec1074 carrying WP_003519433.1 with the expected mass fragmentation shown in panel C

To experimentally validate alcohol acetyltransferase activity (e.g., alcohol + acetyl-CoA → ac(r)yl acetate + CoA), WP_003519433.1 was N-terminus His-tagged and expressed in *E. coli*. Western blot of the purified protein indicated successful expression of WP_003519433.1 (Fig. 7a). This protein was then screened against a library of linear C2-C10 alcohols for acetyltransferase functional activity, but no activity was observed. Interestingly, the LL1210_C1 cluster was enriched for proteins involved in KEGG module M00022, which is part of the shikimate pathway that converts phosphoenolpyruvte and erythrose-4P to chorismate. Overall, the shikimate pathway is involved in the synthesis of aromatic amino acids, which can be used in the production of aromatic alcohols [43]. Further screening revealed that WP_003519433.1 has activity toward the aromatic alcohol 2-phenylethyl alcohol, both in vitro (data not shown) and in vivo (Fig. 7b and c). The synthesized 2-phenylethyl acetate confirmed WP_003519433.1 as an alcohol acetyltransferase. As this enzyme is active toward aromatic alcohols, it likely belongs to EC 2.3.1.- and is different from EC 2.3.1.84 that has substrate specificity toward short-chain alcohols [44–47]. To elucidate the physiological role of WP_003519433.1, further investigation will focus on characterization of *C. thermocellum* that overexpresses and downregulates this enzyme under various conditions.

## Discussion

Despite improvements in gene annotation procedures, a large percentage of genes remain annotated as PUFs in commonly used genome repositories [19, 20]. Although current computational pipelines for functional prediction tools based on sequence homology are powerful [14–16, 24, 41, 42], they are limited to the currently known protein sequence space present in databases and assume sequence similarity implies functional similarity [48]. A protein which differs significantly from any known protein sequence may present challenges to current functional prediction tools. While direct characterization experiments are one option, these are often low throughput. Other methods based on the concept of guilt-by-association (GBA), such as co-expression analysis, may be used to predict putative functions on omics-scale data [29]. Hypothesized putative functions can serve as the basis for further characterization experiments, particularly in identifying the types of experiments needed to confirm a particular function.

To this end, we performed a comprehensive analysis of PUFs in two distinct strains of *C. thermocellum* using a combination of expression analyses (e.g., co-expression and differential expression analyses), evolutionary analysis (e.g., coevolution analysis, gene tree estimation), structural modeling, and sequence homology-based function predictions to identify putative functions for PUFs, with a focus on those potentially related to cellulose degradation, redox balance, and ethanol production. A total of 344 PUFs were measured via LC–MS/MS. Differential expression information and co-expression clusters were generated using proteomics data from two strains of *C. thermocellum* (*Δhpt* and LL1210). Proteins that were differentially abundant across the strains showed clear enrichment of particular functions, such as GO:0016730 (oxidoreductase activity acting on iron–sulfur proteins as donors), which was up-regulated in the LL1210. As many PUFs demonstrated differential expression consistent with proteins of known function, it is likely at least some of these PUFs play roles in these functions under GBA. Importantly, strain LL1210 is an experimentally evolved strain originating from a strain with gene deletions in pathways that compete with ethanol production. It has also been observed that the parent strain of LL1210 is noted for having perturbed redox metabolism [49], Based on previous work, we expected PUFs with potential functional roles in ethanol production and redox metabolism to show differential (co-)expression or temporal patterns relative to the wild-type *Δhpt*, as was observed in this study.

Similar to our differential expression analysis, co-expression analysis identified many clusters containing PUFs in both strains. These clusters were often enriched in various GO and/or KEGG terms, including those related to redox balance, ethanol production, and cellulose degradation. Coevolution analysis identified as subset of PUFs which appear to coevolve with proteins involved in cellulose degradation.

Finally, operon information was also obtained for *C. thermocellum* from the DOOR database [39], which indicates shared regulatory elements of PUFs with proteins of known function, providing another form of GBA. GBA evidence was combined with sequence homology-based information, including domain prediction, structural modeling, and phylogenetic gene tree analysis to hypothesize putative functions for PUFs. These are not meant to serve as official annotations; however, they help to narrow down the list of PUFs with possible interesting putative functions. These selected candidate PUFs can then be validated by other experimental methods, such as gene knockout experiments for phenotype perturbations. Given the evidence presented here, it seems clear that further characterization of PUFs will be a critical for engineering *C. thermocellum* to improve biofuel production.

Importantly, our combination of GBA approaches with sequence homology-based functional/structural prediction identified a putative alcohol acetyltransferase for further experimental characterization. Although co-expression support for this function was modest, it was strongly supported by both sequence homology and gene regulatory information. Experimental characterization revealed that this PUF catalyzes ester formation between acetyl-CoA and aromatic alcohols. While other PUFs had stronger overall evidence, this PUF was chosen for further characterization in part due to a straightforward experimental path for validation. A major challenge for targeted experimental characterization of proteins is the ability to induce a phenotype when experiments are performed in vivo. Without a clear, detectable phenotype, such experimental validations are difficult to achieve.

The *Δhpt* and LL1210 co-expression analyses were based on protein abundance data across 3 and 4 time points, respectively, each with 4 replicates. A larger number of samples would likely result in clusters with clearer functional groupings based on co-expression patterns, as previously described [50]. Despite modest statistical power here, many clusters served as solid evidence for hypothesized functions of PUFs. Further work focused on putative functional identification of PUFs should incorporate more publicly available proteome measurements (with appropriate normalization for different mass spectrometers, label-free quantification methods, etc.) and/or measurement of more samples, ideally varying over a large number of possible growth states and conditions.

Various *in-silico* approaches were employed to complement our analyses based on protein abundance measurements. Many of the sequence homology-based functional annotation tools provided consistent functional information. Although this information was often redundant, suggesting that only one of the tools may be needed, consistent results across tools provide confidence in the predicted function or domains, helping to eliminate possible false positives. Coevolution analyses have also been used previously to test for functional relationships between proteins [34, 51]. Although these in-silico approaches are useful on their own, these approaches are built on their own assumptions, particularly that sequence similarity implies functional similarity, which may not hold over large phylogenetic distances. GBA approaches based on experimental measurements provide another layer of functional information that, while providing less direct functional information, can provide greater confidence in the results of *in-silico* analyses (and vice-versa).

Although some PUFs were highly abundant in both strains, most were low abundance proteins on average (Fig. 2d). Why might PUFs tend to be lower abundance proteins? One possible reason is lower abundance proteins tend to accumulate nonsynonymous substitutions at a faster rate than high abundance proteins [52–54]. We speculate that this could present greater challenges for functional prediction via sequence homology, particularly for species which are relatively distant from better functionally characterized species (e.g., yeast, *E. coli*, mice). To the best of our knowledge, no study has systematically investigated if sequence homology-based predictions perform better on highly expressed genes in which selective constraints on sequence evolution are expected to be stronger, on average. Another, and we believe more likely, reason is a bias towards focusing research efforts on proteins which are more abundant, under the assumption that higher abundance proteins tend to be more important for a species.

Here, we employed both co-expression and phylogenetic analyses of correlated presence/absence of genes as GBA methods for determining functional roles of PUFs. Another option is to examine coevolution of protein abundances across species. Previous work has found that functionally related proteins coevolve at the level of gene expression [55–58]. Such methods could be used to test for functional relationships of PUFs; however, this approach requires the PUFs to be conserved across species, making it most applicable to conserved DUFs. Notably, most of the previous analyses have used codon-based proxies of gene expression (e.g., the Codon Adaptation Index [59]) or data based on RNA-Seq. To date, no work has examined coevolution of protein abundances, even though some evidence suggests that protein abundances are more conserved across species compared to mRNA abundance [60].

## Conclusions

Here, GBA and sequence homology-based approaches were combined to identify putative functions for proteins of unknown function (PUFs) in *C. thermocellum*, with a specific focus on PUFs possibly related to cellulose degradation and ethanol production. One PUF tentatively characterized by our GBA approach, WP_003519433.1, was confirmed experimentally to be an alcohol acetyltransferase. As part of this analysis, a table (Additional file 8) is provided which summarizes the various lines of evidence accumulated for the PUFs in this study. The amount of evidence for any given PUF varies. For example, 216 of the 344 PUFs detected via LC–MS/MS fell into at least one cluster enriched in at least one GO term. For 285 of these 344 PUFs, eggNOG-mapper, PANNZER2, InterProScan, and/or BlastKOALA identified an annotation, although many of these are non-specific or indicate that the protein is uncharacterized. We expect that this table will be of significant interest to the bioenergy research community who may be eager to investigate PUFs of potential interest for further characterization in *C. thermocellum*. The different analyses presented here can easily be applied to other microbes of interest. All functional/structural prediction tools are publicly available, many with easy-to-use web interfaces. During the course of our study, we identified a few proteins which were annotated in the DOOR database, such as WP_003512015.1, despite being a PUF in Genbank/Refseq. Additional file 8 (specifically, columns “Operon Proteins” and “Operon Functions”) can be referenced for other examples. This highlights that, in some cases, major sequence databases such as Genbank/RefSeq may not provide the most up-to-date information. We are likely not the first to note this problem but given the significance of databases like RefSeq for modern biological research, our work supports the need to more effectively keep these databases up-to-date.

## Methods

### Bacterial strains and culture conditions

*Clostridium thermocellum* strains DSM 1313 *∆hpt* [4] and LL1210 [11] were used in this study.

 Strains *∆hpt* and LL1210 were each grown for 30 and 93 h, respectively, inside a Coy anaerobic chamber (Coy Laboratory Products, Grass Lake, MI) under 85% N_2_, 10% CO_2_, and 5% H_2_ gases at 55 °C in quadruplicate 500 mL (total vessel capacity 1L) cultures in MTC5 media [61], along with 5 g/L cellobiose supplemented with 2 mM sodium formate. Formate supplementation in minimal medium improves growth of *C. thermocellum* mutant strains that lack pyruvate-formate lyase (*pfl*) by improving C_1_ metabolism [49], and LL1210 has *pfl* deleted. Samples for proteomic analyses were collected in 50 mL aliquots for timepoints corresponding to early-log, mid-log, and late-log of growth for both strains. Growth phases were determined by optical density values at each timepoint plotted for a growth curve. Additional samples were collected for the lag phase of growth for a total of four sampling events for strain LL1210. Cells were centrifuged (3600×*g*) in 50 mL tubes for 10 min, immediately quenched with liquid nitrogen, and the supernatants were discarded. The samples were then stored at − 80 °C until protein isolation and proteomic analysis.

### Proteome analyses using LC–MS/MS

The *∆hpt* and LL1210 strains of *C. thermocellum* were proteolytically digested (trypsin) for nano-LC–MS/MS analysis. An automated 2D LC–MS/MS analysis was carried out for the peptide samples using an Ultimate 3000 connected in-line with a QExactive Plus mass spectrometer (Thermo Scientific). A triphasic MudPIT back column (RP-SCX-RP) was coupled to an in-house pulled nanospray emitter packed with 30 cm 5 µm Kinetex C18 RP resin (Phenomenex). For each sample, 12 µg of peptides were loaded and cleaned to remove salts (if any) and was separated and analyzed across two successive salt cuts of ammonium acetate (50 mM and 500 mM), each followed by 105 min organic gradient. LC-resolved peptides were analyzed by data-dependent acquisition (DDA) on the QExactive MS.

### MS database searching, data analysis, and interpretation

A non-redundant database was made by combining GenBank and RefSeq *C. thermocellum* proteome databases. The proteins were grouped at 100% identity using CD-Hit. [62] MS/MS spectra were searched against this proteome database concatenated with cRAP databases (ftp://ftp.thegpm.org/fasta/cRAP) consisting of common contaminants using Tide-search [63] keeping a static modification on cysteine (+ 57.0214 Da), and a dynamic modification to an oxidation (+ 15.9949 Da) of methionine. Tide-search was followed by Percolator [64] with default parameters to assign spectra to peptides (peptide-spectrum matches; PSM). Retention times of each PSM were extracted parsing mzML file with in-house script and MS1 apex intensities were assigned using moFF [65]. The moFF parameters were set to 10 ppm for the precursor mass tolerance, 4 min for the XIC time window, and 1 min (equivalent to 60 s) to get the apex for the ms2 peptide/feature. The peptide intensities from were summed to their respective proteins per sample. Protein intensities were then normalized by protein length and overall abundance per MS run. Each protein required a minimum of 2 peptide and 2 PSMs to become a valid protein. Thus, the obtained normalized intensities of proteins were considered valid if a protein exists in 2 out of 4 replicates. Protein abundance distributions were then normalized across samples and missing values imputed to simulate the mass spectrometer limit of detection. All raw mass spectra for the proteome measurements have been deposited into the ProteomeXchange repository with the following accession numbers: (MassIVE Accession: MSV000085237, ProteomeXchange accession PXD018407: FTP link to files: ftp://MSV000085237@massive.ucsd.edu, username is MSV000085237, password is PUF123).

### Validation of alcohol acetyl transferase WP_003519433.1

#### Plasmid construction

The plasmid pET_1074 was constructed using restriction endonucleases (NEB, MA, USA) and DNA ligase (NEB, MA, USA) and propagated in *E. coli* TOP10 (Additional file 9: Table S1). The Clo1313_1074 gene (encoding WP_003519433.1) was PCR-amplified using primers HS566 (5′-CTCTGGATCCA ATGAATTATCCTAAAAAAGTGGAATGG-3′) and HS567 (5′-CTCTGAGCTCCTACATGT TTGACACTATTTC-3′). The amplified PCR fragment and plasmid (pETDuet-1) were digested by BamHI and SacI restriction enzymes, ligated together, and transformed in *E. coli* using a heat shock transformation method. Transformed colonies were PCR verified for successful plasmid cloning using the same primers. The constructed plasmid pET_1074 was verified by Sanger sequencing.

#### Protein expression and purification

To express the His-tagged WP_003519433.1 (Clo1313_1074), *E. coli* C41 (DE3) pLysS was used to maximize the protein production with a tight expression regulation [66]. Ec1074 was cultured in 3 mL Lysogeny broth (LB) medium supplemented with 100 μg/mL ampicillin and 30 μg/mL chloramphenicol in a shaking incubator at 37 °C for overnight (~ 16 h). The overnight culture was inoculated in 50 mL fresh LB medium with 10 g/L glucose and the antibiotics in a shaking incubator at 37 °C until optical density (OD) reached ~ 0.4. To induce the recombinant protein biosynthesis, 0.1 mM of isopropyl β-d-1-thiogalactopyranoside (IPTG) was added to the culture followed by overnight incubation at 18 °C. After the incubation, cells were harvested by centrifugation at 4700 rpm for 10 min. Cell lysis and protein purification followed the method described previously with slight modifications [67]. Briefly, the cell pellets were washed twice with Millipore water before cell lysis by B-PER complete bacterial protein extraction reagent (ThermoFisher Scientific, MA, USA). The cell extracts were incubated with HisPur Ni–NTA superflow agarose (ThermoFisher Scientific, MA, USA) in a batch. After the washing and elution, the eluted protein sample was desalted by Amicon centrifugal filter with 10 kDa molecular weight cut-off (MilliporeSigma, MA, USA). The desalted protein was quantified by Bradford assay with bovine serum albumin (BSA) as the reference protein, before enzyme reaction.

#### SDS-PAGE and western blot

The His-tag purified WP_003519433.1 (Clo1313_1074) was qualitatively analyzed by sodium dodecylsulfate-polyacrylamide gel electrophoresis (SDS-PAGE) and western blot. For SDS-PAGE, Novex WedgeWell 14% Tris–Glycine gel was used (cat# XP00145BOX, Invitrogen, CA, USA). For western blot, proteins after SDS-PAGE were transferred to a nitrocellulose membrane and probed with Anti-6x-His-tag monoclonal antibody conjugated with horseradish peroxidase (HRP). The blot was visualized by 1-Step Ultra TMB (Thermofisher Scientific, MA, USA).

#### Screening of acetyltransferase activity

Acetyltransferase activity of WP_003519433.1 (Clo1313_1074) was screened by an in vitro enzymatic assay conducted in a 100 μL total reaction volume [67, 68]. The reaction solution consisted of 50 mM Tris–HCl pH 7.4, 2 mM acetyl-CoA, 0.5 mg of the purified proteins, and various alcohol concentrations, including 100 mM for ethanol, butanol, isobutanol, pentanol, isoamyl alcohol, 40 mM for hexanol, 20 mM for phenylethyl alcohol, and 2 mM for octanol and decanol with 20% DMSO. 100 μL of hexadecane spiked with 10 mg/L n-decane was overlaid to extract esters. The reaction was carried out at 50 °C for 48 h and the hexadecane layer was analyzed by gas chromatography coupled with a mass spectrometer (GC/MS).

For in vivo verification of the acetyltransferase activity toward phenylethyl alcohol, the IPTG-induced Ec1074 whole cell was concentrated to ODs of 2, 4, and 8 in 4 mL M9 defined medium containing 10 g/L glucose, and 1 g/L yeast extract, 0.1 mM IPTG, and 1 g/L 2-phenylethyl alcohol, and 1 mL of hexadecane with 10 mg/L *n*-decane was overlaid. The whole-cell reaction was performed in a 37 °C shaking incubator for 48 h and the hexadecane layer was analyzed to detect 2-phenylethyl acetate by GC/MS.

#### GC/MS analysis to detect esters

GC (HP 6890, Agilent, CA, USA) equipped with a MS (HP 5973, Agilent, CA, USA) was used to detect esters [44–46, 69]. 1 μL sample was injected into the GC capillary column (Zebron ZB-5, 30 m × 0.25 mm × 0.25 μm, Phenomenex, CA, USA) with the splitless mode at an injector temperature of 280 °C. Helium was used as the carrier gas at a flow rate of 0.5 mL/min, and the oven temperature was programmed as 50 °C initial temperature, 1 °C/min ramp up to 58 °C, 25 °C/min ramp up to 235 °C, 50 °C/min ramp up to 300 °C, and 2-min bake-out at 300 °C.

For the MS system, selected ion mode (SIM) was used to detect esters with the following parameters: (a) ethyl acetate, m/z 45.00 and 61.00 from 4.2 to 4.6 min retention time (RT), (b) isobutyl acetate, m/z 61 and 101 from 6.6 to 7.6 min RT, (c) butyl acetate, m/z 61 and 73 from 7.6 to 8.5 min RT, (d) pentyl acetate, m/z 56, 61 and 73 from 8.5 to 10.1 min RT, (e) isoamyl acetate, m/z 61 and 73 from 10.1 to 10.7, (f) hexyl acetate, m/z 61 and 129 from 10.7 to 11.5. min RT, (g) octyl acetate, m/z 61 and 173 from 11.5 to 13.2 RT, (h) *n*-decane, m/z 78, 99, and 170 from 13.2 to 13.5 RT, (i) decyl acetate, m/z 61 and 167 from 13.5 to 13.8 RT, and (j) 2-phenethyl acetate, m/z 61, 104, and 121 from 13.8 to 15.5 min RT.

### Coevolution analysis

Protein orthogroups (i.e. both orthologs and paralogs) and a species tree were determined using OrthoFinder (see Additional file 1: Figure S2 for the species tree and list of species used) [33]. Coevolution analysis was based on the correlated presence/absence of orthogroups across species, similar to phylogenetic profiling, while accounting for the shared ancestry of species. Orthogroups were treated as discrete species traits with a species containing the orthogroup having a value of 1; otherwise, the species was given a value of 0. Orthogroups containing PUFs identified by LC–MS/MS were then paired with all other orthogroups. Phylogenetic analysis of discrete trait evolution was performed using the corHMM R package [70], assuming no hidden states. For each pair of orthogroups, models were fit either allowing for coevolution or forcing independent evolution. The two models were compared using the corrected version of the Akaike Information Criterion (AICc), which corrects for small sample sizes. Orthogroups were considered to be coevolving if the model allowing for coevolution was 2 or more AICc units better than model forcing independent evolution.

### Differential and co-expression analyses

Differential expression analysis was performed using the R package limma [35] using the limma-trend functionality and robust hyperparameter estimation. Proteins with low expression (here, defined as a normalized abundance less than 23) on average across both strains and all time points were excluded due to violations of limma's assumptions. Proteins were considered differentially expressed if they had a Benjamini–Hochberg corrected *p*-value < 0.05. For the mid-log phase in LL1210, we chose to treat LL1210 time point T2 as the mid-log phase based on the PCA (Additional file 10: Figure S1). Co-expression analysis was performed using imputed protein abundances for both the *Δhpt* and LL1210 strains after filtering out proteins with missing protein abundances in more than 50% of the measurements for a given strain. Clusters were generated for each strain using the Python tool clust [38], with further removal of genes showing low variation across time points. Differential co-expression analysis was performed using the R package DCGL [71] using the DCp method [72].

Gene Ontology enrichment was performed using the R package topGO [73]. For differential expression and differential co-expression analysis, the Kolmogorov–Smirnov test was performed using the weight01 algorithm. For co-expression analysis, the Fisher’s exact test was used with the weight01 algorithm. We note that, per the recommendation of the topGO developers, the p values used for our GO enrichment tests were not corrected for multiple-hypothesis testing. This is because the weight01 algorithm violates the assumptions of independence (see topGO vignette) made by FDR control methods such as the Benjamini–Hochberg correction. Analysis of KEGG terms and modules was performed using the R package clusterProfiler [74]. We note that GO enrichment tests and KEGG over-representation tests were performed using the set of proteins detected via the LC–MS/MS measurements as the background.

### Sequence-based functional predictions

In addition to network analysis, other relevant functional features of PUFs were interrogated via a suite of protein sequence homology approaches. All tools were run with default settings unless otherwise stated. To identify possible enzymatic activity, enzyme commission (EC) numbers and KEGG terms were taken from PANNZER2 [41] and BlastKoala [75], respectively. PANNZER2 was run allowing for 80% minimum alignment length, minimum query and subject coverage of 0.6, and a minimum of sequence identity of 0.4. Functional/domain prediction was also performed using eggNOG-mapper [42] and InterProScan [40]. Gene Ontology terms were pulled from PANNZER2, InterProScan, and eggNOG-Mapper. Many proteins still had no GO terms after the initial analysis. These proteins were re-analyzed with PANNZER2 with a minimum query coverage 0.2 and allowing for a minimum alignment length of 0.2, and with eggNOG-mapper with a 0.1 minimum E value. Structural and cellular localization features of PUFs were further interrogated using SignalP [76], TMHMM [77], and Swiss-Model servers to determine relevant structural properties.

### Gene regulatory information

Genes which are under the same regulatory control often serve related functions within the cell. Operon information, including annotations, for *C. thermocellum* was pulled from the DOOR database [39].

### Phylogenetic gene trees

#### Building a local BLAST database using UniProtKB

To examine possible evolutionary relationships of PUFs with proteins of known function, phylogenetic gene trees were created. Homologs for the PUFs of interest were found using blastp in the BLAST + software suite [78, 79]. FASTA files from Swiss-Prot and TreEMBL were downloaded from UniProtKB and used to create a custom protein sequence database. All *C. thermocellum* PUFs and DUFs were queried against the custom database using an E value cut-off of 10E−5. The searches were done in CADES server at ORNL.

#### Multiple sequence alignment using MAFFT

Following the BLAST homology search, detected homologs for each PUF were aligned using the multiple sequence alignment (MSA) tool MAFFT [80], using the auto-feature to automatically select an appropriate alignment strategy for the given query. The estimation of a highly accurate MSA is necessary to have low error rates when computing the phylogenetic gene trees [81] and this was achieved using the automated feature of, the MSA trimming tool trimAl [82].

#### Phylogenetic gene trees using FastTree

FastTree can compute approximately maximum-likelihood phylogenetic trees from MSA involving protein sequences or nucleotide sequences [83]. Phylogenetic genes trees were generated for a protein alignment using the JTT + CAT model, where JTT [84] is a model for amino acid evolution and CAT is the an approximation used to account for the varying rates of sequence evolution across amino acid sites [85]. Phylogenetic trees were visualized using the ggtree R package [86].

## Supplementary Information

**Additional file 1:** **Figure S2.** Species tree estimated by OrthoFinder and used as the tree for the coevolution analysis.

**Additional file 2.** Enriched GO terms of proteins show to be coevolving with PUFs.

**Additional file 3.** GO enrichment analyses of differentially expressed proteins.

**Additional file 4.** KEGG enrichment analyses of differentially expressed proteins.

**Additional file 5:** **Figure S3.** Volcano plots highlighting significantly different PUFs across strains in A) early-log phase, B) mid-log phase, and C) late-log phase.

**Additional file 6:** **Figure S4.** All clusters generated by clust for the *Δhpt* strain.

**Additional file 7:** **Figure S5.** All clusters generated by clust for the LL1210 strain.

**Additional file 8.** Summary of information for each PUF detected by LC–MS/MS.

**Additional file 9: Table S1.** Strains and plasmids used in validation of alcohol acetyl transferase WP_003519433.1.

**Additional file 10: Figure S1.** PCA analysis of protein abundances across strains and time points.

## Data Availability

All raw mass spectra for the proteome measurements have been deposited into the ProteomeXchange repository with the following accession numbers: (MassIVE Accession: MSV000085237, ProteomeXchange accession PXD018407: FTP link to files: ftp://MSV000085237@massive.ucsd.edu, username is MSV000085237, password is PUF123).

## Abbreviations

PUFs: Proteins of unknown function
DUFs: Domains of unknown function
GO: Gene Ontology
GC/MS: Gas chromatography coupled with mass spectrometry
LC/MS: Liquid chromatography coupled with mass spectrometry
RT: Retention time
MSA: Multiple sequence alignment
GBA: Guilt-by-association
