# Supplementary figures and images for: Identification and characterization of proteins of unknown function (PUFs) in Clostridium thermocellum DSM 1313 strains as potential genetic engineering targets
Source: Biotechnol Biofuels. 2021 May 10;14:116. doi: 10.1186/s13068-021-01964-4 (PMC8112048; doi:10.1186/s13068-021-01964-4)

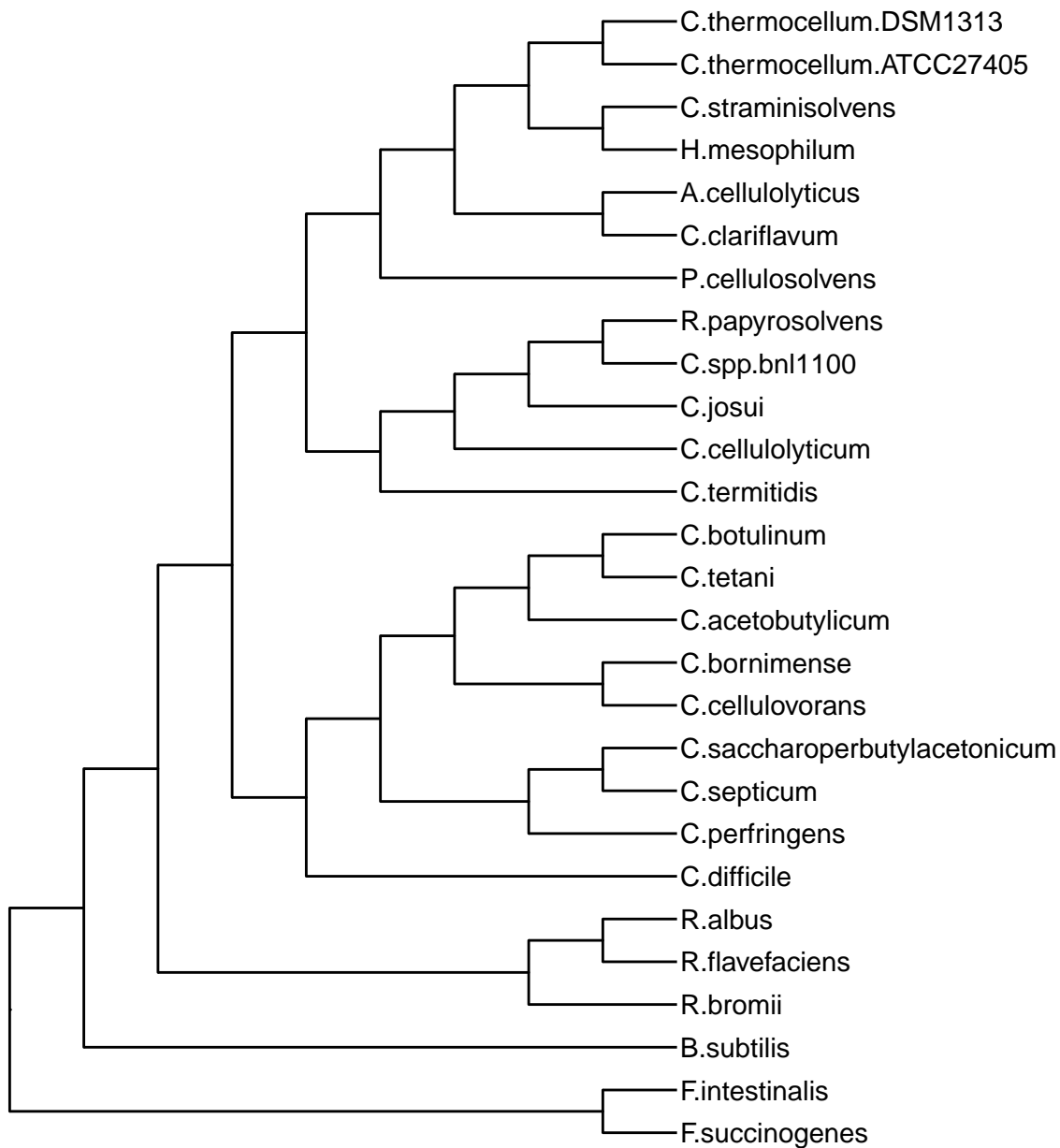

Supplement: Supplementary file 1 — Additional file 1: Figure S2. Species tree estimated by OrthoFinder and used as the tree for the coevolution analysis. [file 13068_2021_1964_MOESM1_ESM.pdf]

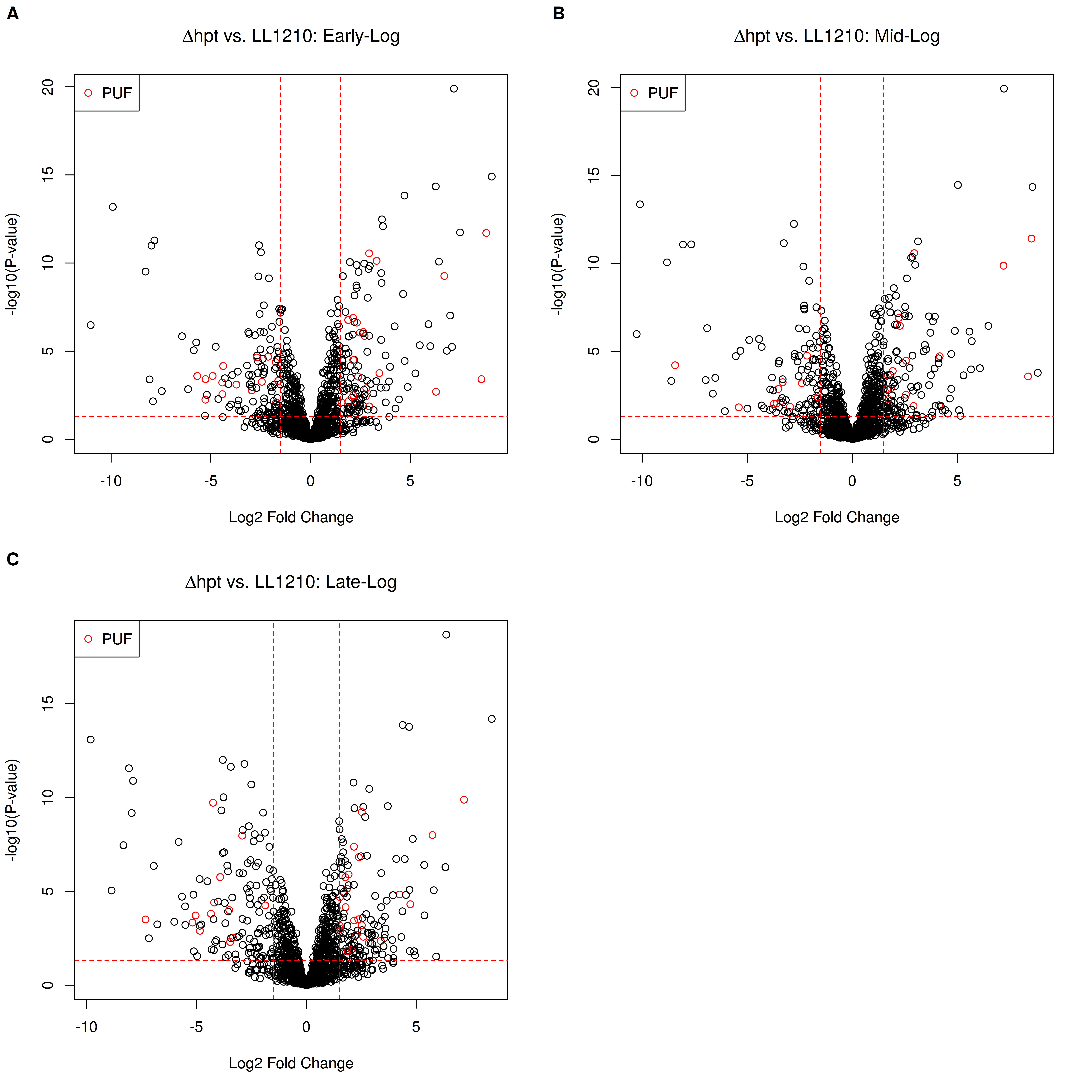

Supplement: Supplementary file 5 — Additional file 5: Figure S3. Volcano plots highlighting significantly different PUFs across strains in A) early-log phase, B) mid-log phase, and C) late-log phase. [file 13068_2021_1964_MOESM5_ESM.png]

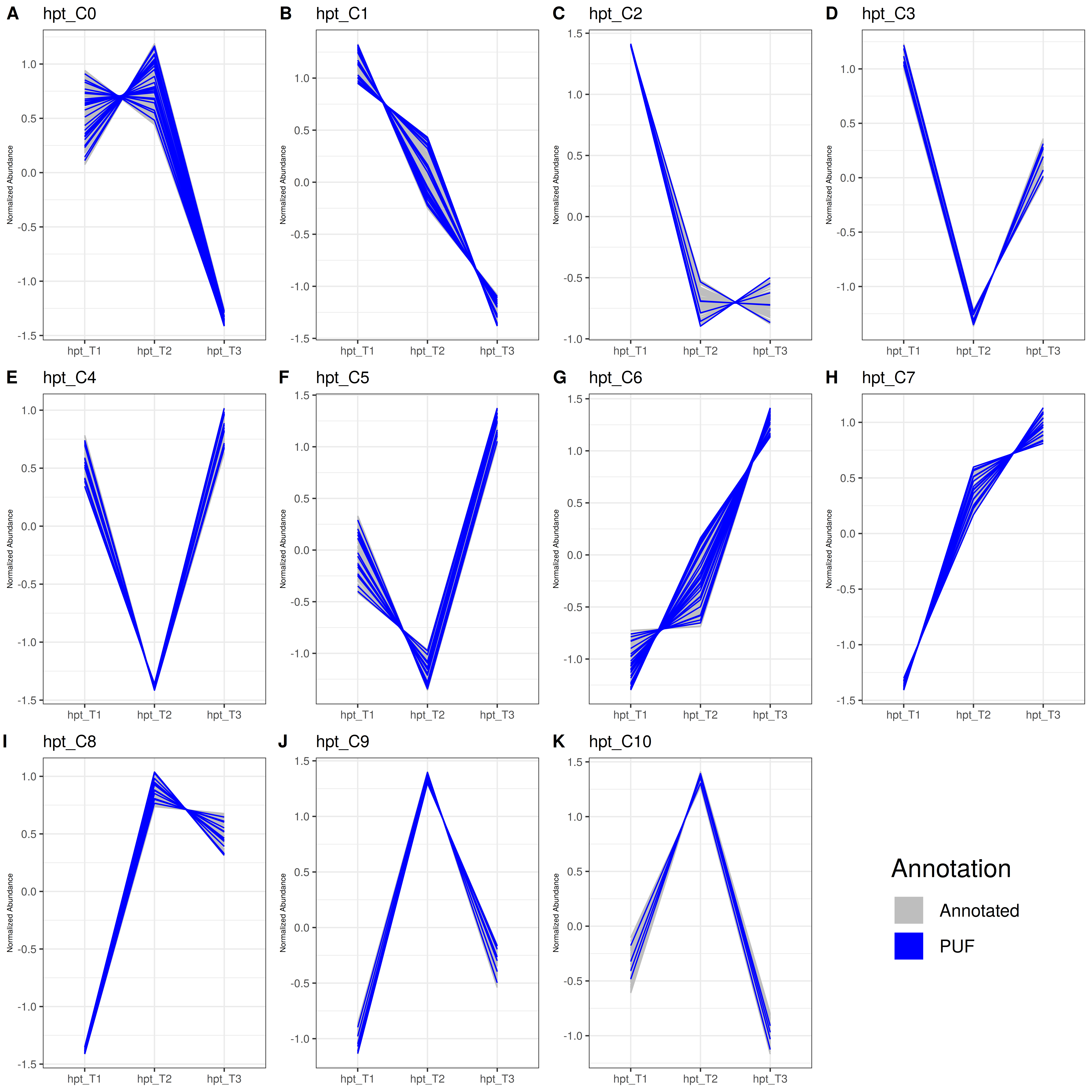

Supplement: Supplementary file 6 — Additional file 6: Figure S4. All clusters generated by clust for the Δhpt strain. [file 13068_2021_1964_MOESM6_ESM.png]

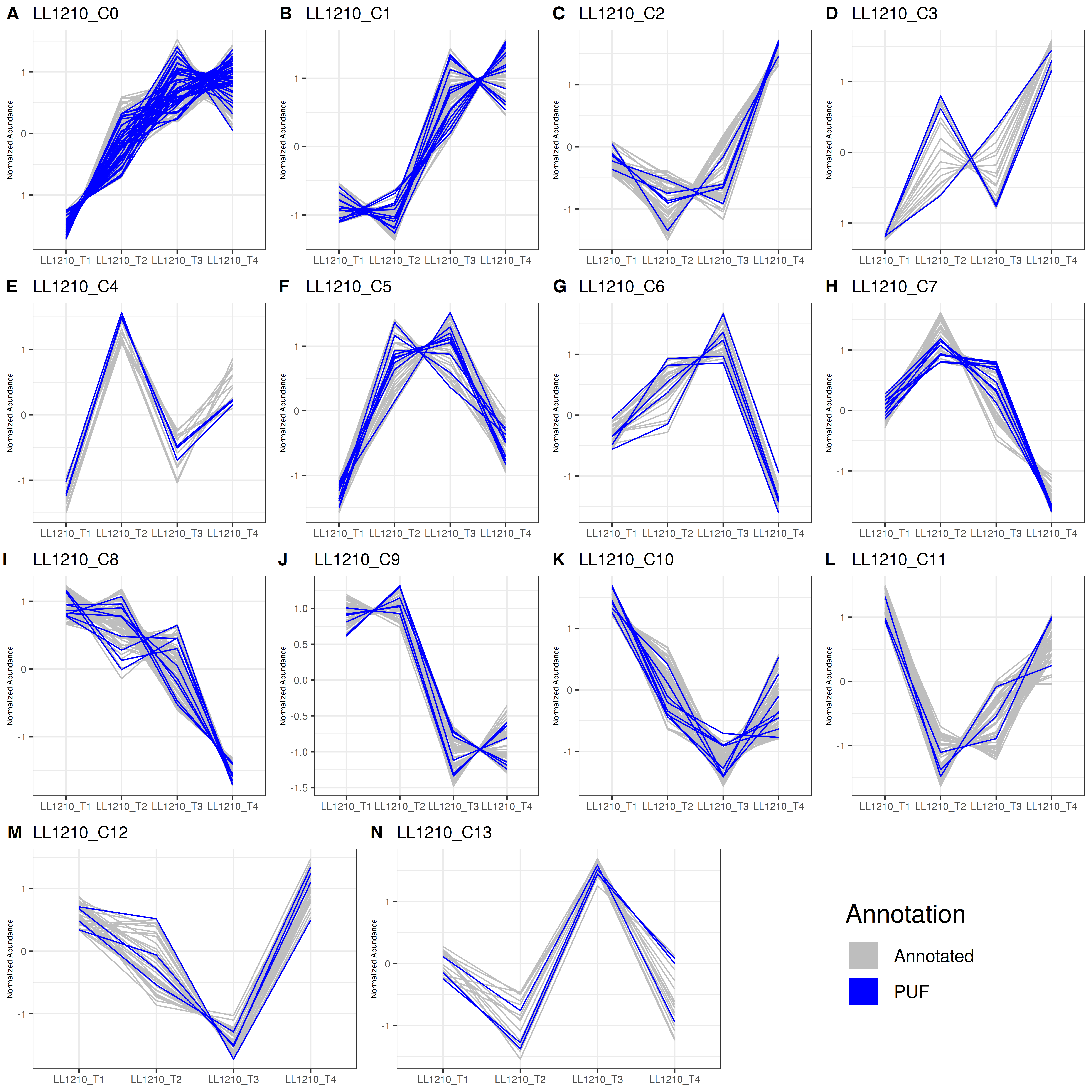

Supplement: Supplementary file 7 — Additional file 7: Figure S5. All clusters generated by clust for the LL1210 strain. [file 13068_2021_1964_MOESM7_ESM.png]

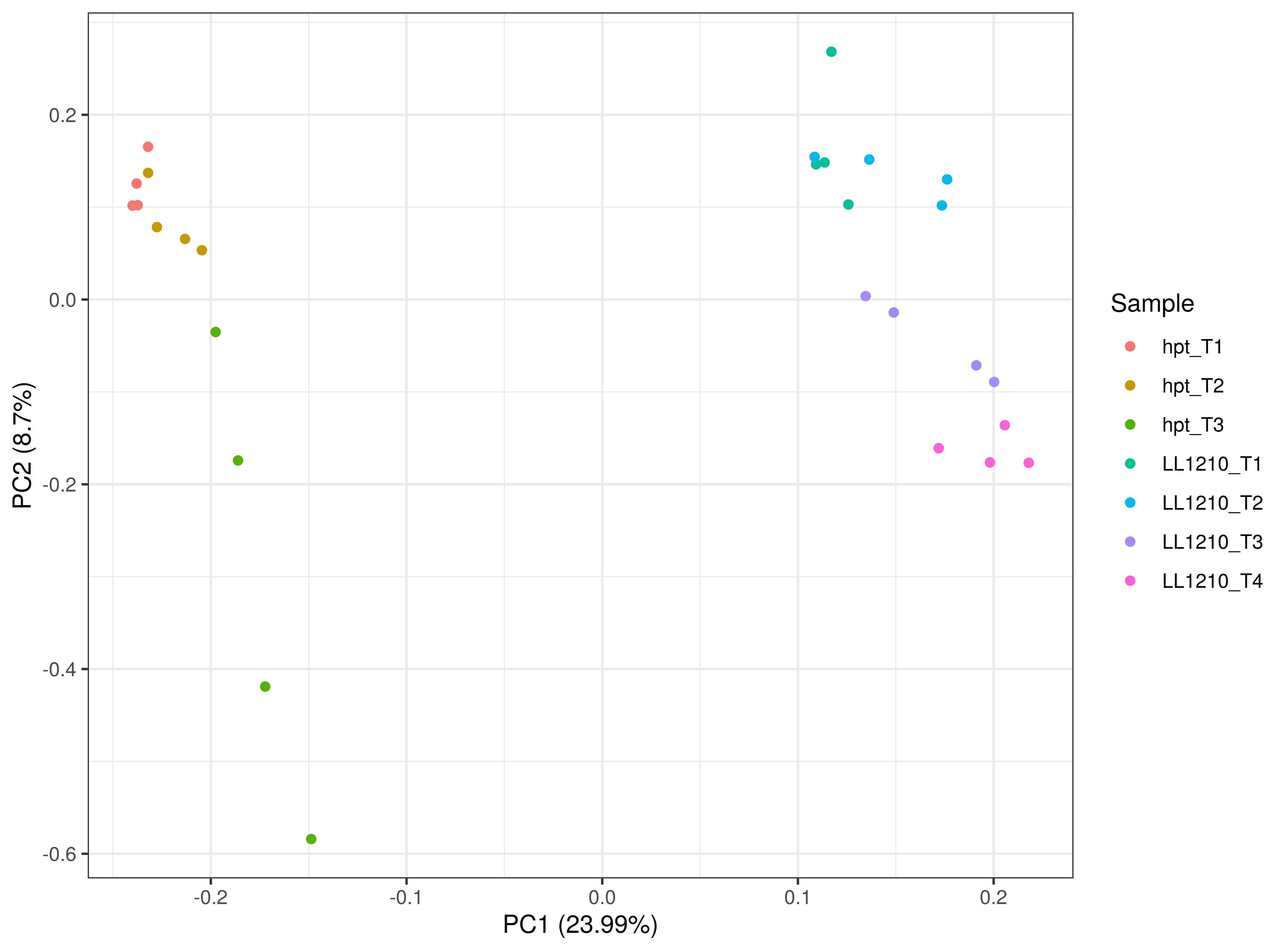

Supplement: Supplementary file 10 — Additional file 10: Figure S1. PCA analysis of protein abundances across strains and time points. [file 13068_2021_1964_MOESM10_ESM.png]
