# Supplementary material for: Identification and characterization of proteins of unknown function (PUFs) in Clostridium thermocellum DSM 1313 strains as potential genetic engineering targets
Source: Biotechnol Biofuels. 2021 May 10;14:116. doi: 10.1186/s13068-021-01964-4 (PMC8112048; doi:10.1186/s13068-021-01964-4)
Supplement: Supplementary file 9 — Additional file 9: Table S1. Strains and plasmids used in validation of alcohol acetyl transferase WP_003519433.1. [file 13068_2021_1964_MOESM9_ESM.docx]

**Supplemental Table 1.** Strains and plasmids used in validation of alcohol acetyl transferase WP_003519433.1

| Strains | Description | References |
| --- | --- | --- |
| *E. coli* TOP10 | F- mcrA Δ(mrr-hsdRMS-mcrBC) φ80lacZΔM15 ΔlacX74 nupG recA1 araD139 Δ(ara-leu)7697 galE15 galK16 rpsL(StrR) endA1 λ- | Invitrogen |
| *E. coli* C41 (DE3) pLysS | F – ompT hsdSB (rB- mB-) gal dcm (DE3) pLysS (Cm^R^) | Lucigen |
| Ec1074 | *E. coli* C41 (DE3) pLysS harboring pET::1074 | This study |
| Plasmids | Description | References |
| pETDuet-1 | *E. coli* expression vector, N-termial 6xHis-tag in MCS1, pBR322 ori, lacI, Amp^R^ | Novagen |
| pET_1074 | pETDuet-1::Clo1313_1074 in MCS1 | This study |
